# A Culturally Appropriate Educational Intervention Can Improve Self-Care in Hispanic Patients With Heart Failure: A Pilot Randomized Controlled Trial

**DOI:** 10.14740/cr346w

**Published:** 2014-07-20

**Authors:** Jill Howie-Esquivel, Kirsten Bibbins-Domingo, Robyn Clark, Lorraine Evangelista, Kathleen Dracup

**Affiliations:** a Department of Physiological Nursing, University of California, San Francisco, CA, USA; b Department of Medicine, University of California, San Francisco General Hospital, San Francisco, CA, USA; c School of Nursing and Midwifery, Flinders University, Adelaide, South Australia, Australia; d Program in Nursing Science, University of California, Irvine, CA, USA

**Keywords:** Heart failure, Self-care, Hispanic, Health literacy, Patient knowledge

## Abstract

**Background:**

Hispanics constitute the largest US ethnic group and have been shown to have more frequent heart failure (HF) hospitalizations than non-Hispanic whites. Disease management programs can reduce HF hospitalizations and mortality by increasing patient self-care, but most programs are limited to patients who speak English. Therefore, we hypothesize that Project Fluido, a culturally appropriate self-care education intervention, will improve self-care behaviors and knowledge in Hispanic patients with HF compared with usual care (UC).

**Methods:**

Project Fluido (N = 42) was a randomized controlled pilot trial over 3 months. Patients in the experimental group (n = 22) received individualized education in Spanish using the “teach-back” method on the following: high salt foods, when to call the physician, when to report weight gain and the use of diuretics. They also received a nurse-initiated phone call every 2 weeks, a script for calling their physician with increased symptoms, a weight scale and a daily diary to complete. The UC group (n = 20) received a scale and written information. Self-care was measured using the self-care heart failure index and knowledge using teach-back scores. Four knowledge topics were included when using teach-back.

**Results:**

Participants’ mean age was 57 ± 14 years, 57% (24) were male, 64% (27) had hypertension, 86% (36) were New York Heart Association Class I-III and 65% (26) had HF with reduced ejection fraction. Participant health literacy scores showed poor health literacy in 31% (n = 13) and 67% (n = 28) spoke Spanish only. Household income was reported as < $20,000 in 93% (n = 39). Self-care and knowledge scores significantly improved (P < 0.04 and P < 0.02, respectively) in the intervention group compared to UC.

**Conclusion:**

The intervention utilized in Project Fluido was a remarkably effective method to improve self-care and HF knowledge in a group of Spanish-speaking HF patients. This improvement is in spite of low physical function, health literacy, acculturation and economic challenges. In addition, teach-back was an effective teaching strategy to improve HF knowledge. Future work is needed to investigate the relationship between increased self-care knowledge, readmissions, and mortality in Spanish-speaking patients with HF.

## Introduction

Heart failure (HF) is a significant public health problem that affects 5.7 million people in the United States [1]. This problem is magnified in the Hispanic population because of the well-documented disparities in health care and the disproportionate cardiometabolic risk burden borne by this population [2, 3]. Outcomes such as rehospitalization rates for Hispanic patients with HF are higher than in Caucasian populations [1]. The language barrier is a significant health care impediment, with over 8 million Hispanics living in the US who do not speak English fluently [4]. Monolingual Spanish speakers have a higher prevalence of cardiovascular risk factors and poorer recognition of cardiac symptoms compared to Hispanics who have English fluency [5].

A review of multiethnic clinical trials conducted in the US between 1995 and 1999 [6] found that Hispanics constituted only 3% of participants yet representing 15% of the general population [7]. Data demonstrating that Hispanics are as willing as non-Hispanic whites to participate in clinical trials suggest that their under-representation in clinical trials is less likely a result of reluctance to enroll, but rather inadequate access to research studies due to language barriers, inaccessibility to study sites and poor information dissemination [8].

Disease management programs focused on improving HF self-care are known to reduce hospitalization rates and mortality [9, 10]. Providing education that enables self/family care is an essential first step for patients with HF. In fact, data demonstrate that the use of a psychoeducational intervention resulted in significantly better knowledge and self-care behavior in rural HF patients [11, 12]. However, few Hispanic patients participated in these studies in which bilingual staff was required. More importantly, the intervention would need to undergo a “cultural magnifying lens.” In other words, the intervention would require consideration of Hispanic cultural values and modification to verify its appropriateness in the context of Hispanic culture. Further, a culturally appropriate intervention would allow examination of long-term outcomes such as rehospitalization and mortality.

In patients from non-English speaking backgrounds, consideration of health literacy is fundamental to improving health outcomes. A successful method for HF patient teaching, termed “teach-back,” takes all literacy levels into consideration [13, 14]. Teach-back was found to be a successful teaching method in a large group of hospitalized multi-ethnic HF patients [13]. No studies to date have been reported on the use of a culturally and health literacy-appropriate educational intervention exclusively for Spanish-speaking patients with HF. The potential for reducing health disparities among Spanish-speaking HF patients remains a significant opportunity for future research.

### Hypothesis

We hypothesized that a culturally and health literacy-appropriate self-care educational intervention would improve self-care behaviors and HF knowledge compared with a usual care (UC) group. In addition, we examined the relationship between psychosocial factors and the outcomes of self-care and HF knowledge.

## Methods

### Research design

The research design of Project Fluido was a prospective, pilot randomized controlled trial (RCT) that followed HF patients over 90 days. This study included two groups, an intervention and UC group with follow-up conducted at 3 months.

### Sample

A convenience sample of 42 patients with HF were enrolled. Inclusion criteria were: 1) ≥ 18 years of age, 2) hospitalized for HF within the previous 12 months, 3) identify themselves as Hispanic in origin, and 4) speak or write in English or Spanish. We included patients who had been recently hospitalized as they are at higher risk for readmission. Exclusion criteria included: 1) cognitive impairment that limited ability to understand and complete questionnaires, 2) severe life-limiting co-morbidity (e.g. life expectancy of less than 6 months), and 3) live in a nursing home (could not participate in self-care activities).

### Procedure

The study was approved by the Institutional Review Board at the University of California, San Francisco (UCSF), Research Committee at San Francisco General Hospital (SFGH) and nationally registered (NCT02083744). Recruitment took place at both UCSF and SFGH between April 2012 and March 2013 until the final sample size was obtained. Eligible patients were first introduced to the research study by other healthcare providers (cardiologists or nurse practitioners), and then interested patients were provided details of the study and enrolled by the research nurse. Patients who presented to the cardiology clinic or were being discharged from the hospital were recruited. The process of designing the study as culturally appropriate took place over 6 months prior to the initiation of the pilot study. Experts in HF care, Hispanic culture, lay people and nursing convened to design the study and materials with details described elsewhere.

After written consents were obtained, patients completed the Mini-Cog test for cognitive function to verify eligibility and completed baseline questionnaires [15]. The research nurse then opened sealed opaque envelopes that contained the assignment to the intervention or UC group. Due to the nature of the study design, the PI or the research nurse could not be blinded to the intervention once group assignment was determined.

Clinical data were collected from medical record review and patients completed the following questionnaires with the research nurse: 1) sociodemographic survey, 2) self-care heart failure index [16, 17], 3) brief symptom inventory subscale for anxiety [18], 4) patient health questionnaire-8 [19], 5) multi-dimensional scale for perceived social support [20], 6) short assessment of literacy for Spanish adults [21], and 7) the short acculturation scale for Hispanics [22].

The intervention consisted of five components (Table 1). The intervention group received a 1-on-1 educational session conducted by a bilingual/bicultural research nurse at the clinic site, the patients’ home, or local cafe depending on patient preference. Patients received the educational intervention via a laptop computer and then teach-back questions were discussed (Table 1). To reinforce the educational intervention content a DVD, memory stick, or paper copy of the presentation was provided to the patient (whichever the patient preferred). Patients received a scale to measure weight, a diary to record weight and symptoms of HF, culturally appropriate no-salt spices and recipes, and telephone follow-up from the research nurse to reinforce the content of the education program every other week two additional times. The research nurse contacted patients every 2 weeks to: 1) reinforce the intervention and use of the diary, 2) discuss weights and symptoms, 3) discuss any unplanned healthcare visits, and 4) provide opportunity to ask questions. Patients were also given a script to use when calling their healthcare provider about increased HF symptoms in Spanish and English. The purpose of the script was to guide them in how to discuss their symptoms, if worse, with their healthcare provider. Patients were called close to the end of their 90-day participation to schedule a follow-up visit at which time four questionnaires (self-care heart failure index, brief symptom inventory subscale for anxiety, patient health questionnaire-8, multi-dimensional scale for perceived social support) were repeated.

**Table 1:** Components of the Intervention

1-on-1 education	Presentation on laptop & written materials
Scale, spices and recipes	Daily weights done upon awakening No-salt, chili-based spices Low-salt Latin-American recipes in large text format
Script	Word for word description of how to talk with their health care provider in English and Spanish
Diary/calendar	Patients recorded daily weights and symptoms
Telephone follow-up	Warning signs for action

Patients in the UC group were given a scale and the educational materials (without the 1-on-1 education). UC patients were not instructed on how to use the scale. Since most patients were from low socio-economic backgrounds, the researchers felt that it was unethical to withhold scales. The UC group did not receive spices, recipes, script or nurse follow-up phone calls.

All patients were given a $50 local grocery store gift card upon completion of the questionnaires. Clinical outcomes were examined in this study by capturing adverse events such as all cause morbidity and mortality within 90 days through electronic medical record review and from patient phone calls.

### Outcome measures: self-care and HF knowledge

Self-care management skills were measured using the self-care heart failure index [16, 17]. This instrument is a 22-item well-validated instrument that measures self-care with three subscales: management, maintenance and confidence. The instrument was validated in Spanish speakers and items are scored in a 0 - 100 range [17].

The measurement of HF knowledge took place during the process of teach-back. Teach-back is a health-literacy appropriate method of patient teaching where the educator teaches the patient in plain language and then asks the patient to teach-back the information that had been presented to them [13, 23]. The concept underpinning teach-back education involves asking patients to restate information that has been presented to them. Teach-back education can serve as a method of education and a tool to assess learning.

HF-specific education was provided to the patients and included information related to: physiological function of the heart, rationale for fluid and sodium restrictions, importance of adherence to pharmacological therapies, rationale for weighing daily, signs and symptoms warranting provider notification, and how to talk to their provider. Patient’s family members, caregivers, and/or support persons were also educated when available. Learning was assessed using four teach-back questions at the conclusion of the education session (Table 2). The four knowledge topics included educational content related to: high salt foods, when to call their healthcare provider, when to report weight gain and the use of diuretics. Patients with incorrect responses were provided further education until understanding was achieved.

**Table 2:** Teach-Back Questions

Teach-back question	Area of focus
What is the name of your water pill?	Medications
How much weight gain would you want to report to your MD?	Self-monitoring skills
What high-salt foods do you need to avoid/be aware of?	Diet modification
Please name 3 - 4 symptoms or warning signs of when you want to call the MD?	Warning signs for action

At the conclusion of the teaching each patient was asked four teach-back questions.

### Psychosocial and literacy measures

Acculturation and health literacy were only measured at baseline because it was unlikely that they would change within 90 days. Acculturation was measured using the short acculturation scale for Hispanics that allows researchers to categorize study participants into high or low acculturation levels using four questions [22]. Validity and reliability are high and the scale has been tested in Mexican Americans, Puerto Ricans, Central and South Americans [22]. Health literacy was measured using the short assessment of health literacy for Spanish-speaking adults (SAHLSA-50), which incorporates word recognition and comprehension using multiple choice questions [20]. This valid and reliable instrument was found to be associated with physical health in Spanish-speaking adults [20].

Depression was measured with the eight-item personal health questionnaire-8, a brief and valid tool used for depression screening [19]. Scores range from 0 to 24 and a score of 10 or greater suggests clinical depression. The personal health questionnaire-8 has well-established validity, sensitivity and specificity [19]. Anxiety was measured using the six-item anxiety subscale of the brief symptom inventory and measures state anxiety [18]. Scores range from 0 to 4 and the overall score is the average score of all items with higher scores indicating higher anxiety. It has demonstrated reliability and validity in cardiac patients [18]. Social support was measured using the validated 12-item multidimensional scale of perceived social support [20]. Perceptions about support from family, friends and a significant other were assessed with scores ranging from 7 to 84 where higher scores indicated higher levels of perceived support.

### Statistical analysis

Descriptive statistics, means and standard deviations (SDs) for continuous variables, and frequencies and percentages for categorical variables were provided for all sociodemographic and clinical variables. Study group differences in sociodemographic and clinical characteristics were examined with Chi-square and Student’s *t*-tests analyses. All data were analyzed with intention to treat. Missing data were assumed to be missing completely at random. SPSS 21.0 was used for all analyses.

In order to estimate the necessary sample size, power analyses were performed utilizing data from a previous study [9]. It was hypothesized that the intervention group would show more improvement in HF self-care behavior and HF knowledge than the UC group. For self-care, which was the outcome determining sample size for this pilot study, the difference between the intervention and control groups was 1.0 with a common SD of 1.15, therefore the estimated effect size was d = 0.87. Based on an estimate of the effect size being at least d = 0.87, in order to achieve power of 0.80 at an alpha level of 0.05, 22 subjects per group (total sample of 44) were needed to find statistical significance.

To compare the effect of the intervention to UC on HF knowledge and self-care behaviors, a repeated measures analysis was used with a linear mixed models approach. This analysis allows for testing the main effect of group, main effect of time and the group-by-time interaction. Linear regression was used to examine predictors of self-care and teach-back knowledge. Significance was defined as P < 0.05 and data are presented as means ± SDs where appropriate.

## Results

### Characteristics of study participants

**Figure 1:**
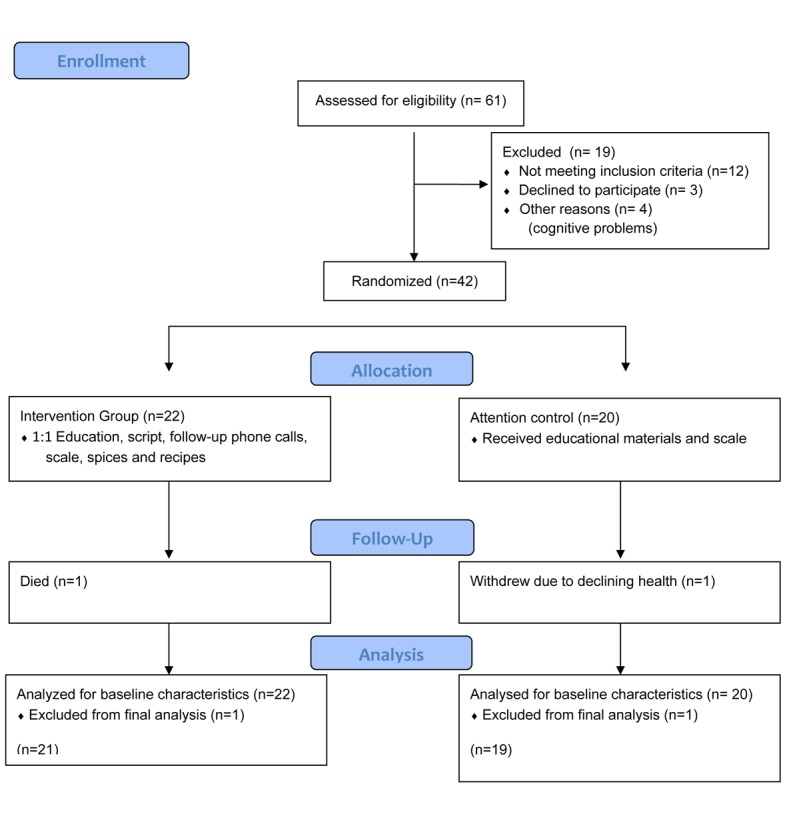
Patient screening and recruitment.

Forty-two patients were recruited to participate and completed baseline questionnaires; however, one patient withdrew and one died before the study end. Figure 1 displays patient screening and recruitment according to the CONSORT guidelines [24]. No differences in sociodemographic or clinical characteristics (including ED or hospitalizations) were found among groups except with depression levels, where the UC group had higher baseline levels of depression (Table 3). Overall, the sample mean age was 57 ± 14 years (range 25 - 84), 57% were male, and 57% unmarried. Poor health literacy was present in 31%, low acculturation scores in 52%, and 67% spoke Spanish only. Financial resources were limited with 93% of participants who reported an income of < $20,000 and 38% lived with four or more in the home.

**Table 3:** Baseline Sociodemographic, Clinical and Psychological Characteristics (N = 42)

	Usual care group (n = 20)	Intervention group (n = 22)	P
Age, years (range 25 - 84)	53.7 ± 12.3	60.7 ± 14.8	0.11
Gender			0.21
Female	11 (55.0)	7 (31.8)	
Male	9 (45.0)	15 (68.2)	
Low acculturation score^a^	13 (41)	9 (60)	0.10
Living arrangement			0.39
Lives alone	3 (15.0)	2 (9.1)	
Lives with 1 - 3 others	12 (60.0)	9 (40.9)	
Lives with 4 - 7 others	3 (15.0)	8 (36.4)	
Lives with 8 or more	2 (10.0)	3 (13.6)	
Annual salary			0.23
< 20,000	19 (95.0)	20 (90.9)	
20,000 - 40,000	0 (0)	2 (9.1)	
40,000 - 75,000	1 (5.0)	0 (0)	
Relationship status			1.0
Married	9 (45.0)	9 (40.9)	
Not-married	11 (55.0)	13 (55.1)	
Sahlsa^b^ score	40.4 ± 6.7	42 ± 6.4	0.40
Clinical characteristics			
NYHA classification			0.49
NYHA I	0 (0)	1 (4.5)	
NYHA II	5 (25.0)	9 (40.9)	
NYHA III	12 (60.0)	10 (45.5)	
NYHA IV	3 (15.0)	2 (9.1)	
Hypertension	12 (60.0)	15 (68.2)	0.75
Diabetes	8 (40.0)	10 (45.5)	0.66
Ejection fraction < 40%	6 (30.0)	8 (40.0)	0.74
Taking ACE-I	11 (45.8)	13 (54.2)	1.0
Taking ARB	1 (5.0)	3 (13.6)	0.60
Psychosocial characteristics			
Depression^c^	10.8 ± 5.89	6.73 ± 5.85	0.03
Anxiety^d^	1.13 ± 1.09	0.62 ± 0.74	0.09
Social support^e^	64.7 ± 14.5	64.1 ± 13.1	0.88

Data are presented as mean ± SD, n (%), P < 0.05. ^a^Based on short acculturation score for Hispanics (less than 2.99 is less acculturated). ^b^Sahlsa, short assessment of health literacy for Spanish adults. ^c^Based on patient health questionnaire-8 (range 0 - 24, 10 or greater indicates depression). ^d^Based on brief symptom inventory subscale for anxiety (range 0 - 4, higher score indicates anxiety). ^e^Based on multi-dimensional scale of social support (range 7 - 84, 69 - 84 indicates high perceived levels of support). NYHA: New York Heart Association classification (range I - IV); ACE-I: angiotensin converting enzyme-inhibitor; ARB: angiotensin receptor blocker.

Clinical characteristics included 64% who had hypertension, 64% were New York Heart Association (NYHA) Class III/IV and 65% had HF with reduced ejection fraction. Psychosocial measures revealed mean baseline depression scores of 8.6 ± 6.15 (range 0 - 27 where depression is present when scores are ≥ 10), mean anxiety scores of 0.87 ± 0.95 (range 0 - 4 where higher scores indicate more anxiety) and social support scores of 64 ± 14 (range 7 - 84 where higher scores indicate higher levels of perceived support). Both the intervention and UC groups did experience improvements in depression and anxiety scores but the differences were not significant (Table 4).

**Table 4:** Self-Care, Teach-Back and Psychosocial Scores by Group

	Usual care group (n = 20)	Intervention group (n = 22)	P
Teach-back scores			0.04
Mean	3.8	3.6	
Baseline	3.9	4.0	
Final range	(3.8 - 3.9)	(3.9 - 4.1)	
Self-care management^a^			0.02
Mean	58.9	49.2	
Baseline	58.0	81.0	
Final	(41.9 - 74.2)	(62.7 - 99.4)	
Self-care maintenance^a^			0.88
Mean	56.8	63.6	
Baseline	70.0	77.7	
Final	(61.3 - 78.6)	(69.1 - 86.1)	
Self-care confidence^a^			0.79
Mean	68.5	73.5	
Baseline	70.8	74.0	
Final	(61.8 - 80.3)	(64.7 - 83.4)	
Depression^b^			0.69
Mean	10.8	6.7	
Baseline	9.3	6.1	
Final	(5.7 - 12.8)	(2.6 - 9.6)	
Anxiety^c^			0.26
Mean	1.13	0.62	
Baseline	0.83	0.71	
Final	(0.35 - 1.3)	(0.23 - 1.9)	
Social support^d^			0.57
Mean	64.7	64.1	
Baseline	64.2	65.6	
Final	(56.8 - 71.5)	(58.5 - 72.8)	

^a^Based on the self-care heart failure index. ^b^Based on patient health questionnaire-8. ^c^Based on brief symptom inventory subscale for anxiety. ^d^Based on multidimensional scale of social support. CI: confidence interval.

### Self-care and knowledge

Mean baseline self-care management scores were 56 ± 23, self-care maintenance 60 ± 18 and confidence 71 ± 20 (all subscales range 0 - 100 with higher scores indicating better self-care). In the intervention group, self-care management scores significantly improved from a mean of 49 to 81 (P = 0.02). Self-care maintenance improved in both groups, but was not statistically different. Self-care confidence did not improve. Anxiety was found to be a predictor of self-care maintenance (P = 0.03, CI: -15.5, -0.84), while depression and social support were not. For every one-unit increase in anxiety, there was an eight-point decrease in self-care management (Table 5). Depression scores did improve in both groups; however, baseline scores were significantly different between the groups (higher in UC).

**Table 5:** Psychosocial Predictors of Self-Care and Heart Failure Knowledge (N = 42)

Baseline	SE	P	Exponent B	95% CI
Self-care maintenance^a^				
Depression^b^	0.56	0.58	0.32	-0.82, 1.45
Anxiety^c^	3.63	0.03	-8.20	-15.5, -0.84
Social support^d^	0.19	0.08	0.35	-0.06, 0.75
Teach-back knowledge				
Depression	0.017	0.284	0.018	-0.02, 0.05
Anxiety	0.108	0.327	-0.107	-0.33, 0.11
Social support	0.01	0.11	0.01	-0.00, 0.02

SEM: standard error of mean (P < 0.05). CI: confidence interval. ^a^Based on self-care heart failure index. ^b^Based on patient health questionnaire-8 depression instrument. ^c^Based on brief symptom inventory subscale for anxiety instrument. ^d^Based on multi-dimensional scale of social support instrument.

Mean baseline teach-back scores were 3.69 + 0.51 (range 0 - 4 with higher scores indicating better knowledge). In the intervention group, teach-back scores significantly improved from a mean of 3.6 to 4.0 (P = 0.04). Mean time spent teaching the intervention group was 78 ± 18 min. There were no significant psychosocial predictors of teach-back scores. The effect size of the intervention for comparing the intervention to UC group was calculated on self-care scores. There was a large effect size with Cohen’s d = 0.89.

## Discussion

These pilot data demonstrate that a culturally appropriate educational intervention improved self-care behaviors and HF knowledge in comparison to a UC group in the first study of its kind. In addition, anxiety was a predictor for self-care management.

Few studies address the association between minority status and self-care management in cardiovascular disease. One group of investigators examined the association between minority status and self-care confidence in recently hospitalized cardiac patients [25]. Hispanics had the lowest confidence levels to self-manage in comparison to black or white patients. However, when health status and socio-economic circumstances were controlled for, the association was no longer apparent, thus illustrating the importance of clinical and economic conditions on health. In our study, we measured self-care confidence and found that our intervention did not significantly increase self-confidence. However, we found that self-care management did increase, calling into question the exact relationship between self-care confidence and self-care management. If self-care confidence skills increase, do self-care management skills, or the behaviors of self-care change?

Caldwell and colleagues tested a similar educational intervention in a small group of rural HF patients and found that self-care and HF knowledge improved [11]. In a large group of rural HF patients, investigators found improvements in self-care, HF knowledge and cardiac mortality after testing an educational intervention in two doses, LITE and PLUS where the LITE group demonstrated improved cardiac mortality [12]. Our intervention key elements (education, scale, script and nurse phone calls) were similar to the LITE group and our results are comparable although our study was not powered to test outcomes such as mortality and hospitalizations.

Despite the numerous endorsements from national organizations such as the Heart Failure Society of America to provide patient education, no studies have been conducted to compare the efficacy of different patient teaching methods. Several investigators have demonstrated that teach-back is an effective strategy for patient teaching [13, 14]. However, few studies have linked patient teaching methods to improved patient outcomes. A health literacy sensitive approach to patient education demonstrated improvement in HgA1c levels in one small study [23]. In the only RCT that tested teach-back on patient outcomes known to the authors, asthmatic patients had improved asthma knowledge and fewer 30-day acute health-related events [26]. In our study teach-back was associated with improved HF knowledge, although our sample size was not large enough to test clinical outcomes.

Psychological symptoms such as depression have been found to correlate with low self-efficacy in chronic illness self-management interventions [27]. Sin and colleagues assessed the prevalence and personal characteristics that predict depressive symptoms in Hispanics with HF after a telephone case management intervention [28]. Depressive symptoms were highly prevalent and acculturation, co-morbidity and NYHA class were significantly related to depressive symptoms. In our study, depression symptoms improved in both the control and the intervention groups. The cause of the improvement in symptoms is unclear, but may be related to the Hawthorne effect. Anxiety, but not depression significantly predicted self-care management scores. It is known that HF patients experience high levels of anxiety [29], but no data exist to describe anxiety in Hispanic patients with HF. Riegel and colleagues investigated the effect of a 6-month telephone case management program on hospital readmissions and depression in one of the few disease management studies that enrolled Hispanics of Mexican-American origin [30]. The authors found no improvement in HF readmissions, mortality or depression. This study did not include an educational or cultural component to the intervention and a subsequent editorial opined that HF knowledge and patient beliefs may have played a role in this negative study [31].

Limitations may apply in this pilot RCT. First, the small convenience sample may have undermined the external validity of the findings. The sample was predominately male, low-income and with low health literacy. The results may be different in women and populations reflecting greater income and education. Second, the follow-up period of 90 days did not allow for long-term adherence trends or clinical outcomes, particularly given the setting of a chronic disease such as HF. The Hawthorne effect may have been present in this study, especially within the UC group since they received scales and patient education handouts. The scales and handouts may have added unintentional attention that resulted in changes in anxiety and depression scores.

Despite the potential limitations, several positive attributes of this study are present. No research to date regarding culturally appropriate interventions for the largest ethnic group in the US has been previously reported. The feasibility of this intervention has been established with a low attrition rate, and may be generalizable to a wider population. Future research in disease management with underserved populations will help determine the appropriate dosing and content of education and follow-up through long-term studies. Improvements in self-care and knowledge are needed to correspond to long-term clinical outcomes such as rehospitalization and mortality. As our population continues to diversify in ethnicity, race, sex and age, studies are needed to address the key aspects of self-care skill building and patient education.

### Conclusion

Project Fluido was an effective and culturally appropriate intervention to improve self-care and HF knowledge in a group of Spanish-speaking HF patients. The improvement was achieved despite low physical function, poor health literacy, acculturation and economic challenges faced by the participants. In addition, teach-back was an effective teaching strategy to improve HF knowledge. Future work is needed to investigate the relationship between increased self-care knowledge and hospital readmissions and mortality in Spanish-speaking patients with HF.
